# Public Awareness and Recognition of Alarming Symptoms of Post-traumatic Intracranial Hemorrhage: A Cross-Sectional Study

**DOI:** 10.7759/cureus.114924

**Published:** 2026-08-21

**Authors:** Hani A Abozaid, Thikra K Aloufi, Eatezaz M Alnefaie, Amjad F Aldosari, Aljoharah A Alotibi, Saad S Alzahrani, Amr A Abdel-Aziem

**Affiliations:** 1 Department of Family and Community Medicine, Taif University, Taif, SAU; 2 Department of Physical Therapy, Taif University, College of Applied Medical Science, Taif, SAU

**Keywords:** alarming signs, awareness, convulsion, headache, intracranial hemorrhage, trauma

## Abstract

Background and objectives: Early management can greatly reduce morbidity and mortality rates in post-traumatic intracranial hemorrhage (ICH). This study assessed the level of awareness and recognition of the alarming symptoms of post-traumatic ICH in Taif, Saudi Arabia.

Methods: A non-probability convenience sampling method was used to recruit participants aged ≥ 18 years (434 participants). A structured questionnaire was used to determine the risk factors, alarm signs and symptoms, ability to recognize these signs, and participants who experienced head trauma.

Results: The questionnaire demonstrated acceptable reliability (overall Cronbach's α = 0.9, McDonald's ω = 0.9; item-deleted range 0.7-0.9). The mean awareness score was 9.8 ± 5.5, with 37.7% (n=164) of participants showing poor awareness. Most of them recognized convulsions (71.0%, n=308) and loss of consciousness (70.3%, n=305) as alarming symptoms. Awareness did not differ by prior head trauma history (p > 0.05). Males and older age were significant predictors of higher awareness of multiple symptoms (p < 0.05), whereas educational levels showed no consistent association.

Conclusions: There is moderate awareness regarding certain symptoms such as altered levels of consciousness and convulsions. However, notable gaps persist in awareness of several risk factors and child-specific symptoms, including changes in feeding habits, alterations in breastfeeding patterns, and disruptions in sleep routines. The male sex and older age were significantly associated with awareness of several individual post-traumatic ICH warning signs, although these associations should be interpreted cautiously given the study’s convenience sampling, cross-sectional design, and lack of adjustment for multiple comparisons. As the study relied on a non-probability convenience sample recruited through social media, the sample cannot be considered representative of the general population. Enhancing awareness of risk factors and alterations in sleep patterns of children requires training programs for parents, caregivers, and the public.

## Introduction

Intracranial hemorrhage (ICH) is defined as bleeding that occurs in the cranium, affecting the cerebrum or cerebellum parenchyma and sometimes both at the same time, causing devastating events including long-term disability or death if not managed well. There are multiple locations where blood could accumulate in the cranium, of which the most common in trauma settings is the epidural space, creating an epidural hematoma [1]. Excessive bleeding can cause raised intracranial pressure and, as a result, oxygen deprivation of the brain, potentially leading to life-threatening brain injury [2]. Traumatic brain injury (TBI) is a major medical, surgical, and public health issue that affects many victims of trauma of all ages and in all populations. Traumatic brain injury is characterized by a high-energy force directed to the cranium that can be due to many etiologies, such as falls, assaults, gunshot wounds, and road traffic accidents (RTAs). Annually, it affects more than 10 million individuals around the globe, leading to hospitalization and treatment and, in severe cases, death on scene. [3].

Moreover, it is a major contributor to long-term impairment, accounting for 40% to 50% of TBI fatalities. The effect of post-traumatic ICH on mortality and morbidity highlights the significance of early detection for vigorous medical and surgical treatment [4]. Most people experienced post-traumatic ICH and noted that the first symptom is a sudden, painful headache; alterations in the level of consciousness, vision problems, nausea and vomiting, confusion, and severe tiredness are also considered warning symptoms [5]. Nonetheless, patients exposed to head trauma must be evaluated immediately, including a thorough neurological examination according to diagnostic and treatment guidelines. Usually, the diagnostic techniques utilized will depend on the severity of the head trauma and the patient's responsiveness during a Glasgow Coma Scale (GCS) evaluation, imaging tests, and monitoring of intracranial pressure [6].

Head injuries are a major cause of death and disability among young people. The Kingdom of Saudi Arabia accounts for 4.7% of all road traffic deaths [7,8]. In Saudi Arabia, the head and neck were the most harmed body regions, followed by upper and lower extremities due to RTAs, which cause roughly 19 deaths and four injuries per day. Therefore, there is an urgent need for scientific evidence-based information to effectively educate the public about road safety and plan suitable interventions to prevent loss of life and lifelong disability [8].

Despite the high prevalence of head trauma, the community has a poor understanding of its management and risk of complications [9]. Multiple surveys on brain injury in North America and Europe [10-12] suggested that most adults have heard of concussion or 'head injury'. However, very few can correctly identify the post-traumatic ICH symptoms that warrant emergency evaluation. Moreover, there is an underestimation of the risk of delayed deterioration following an apparently minor trauma. These reports further showed that awareness of medical risk factors, including older age, use of anticoagulants or antiplatelet agents, uncontrolled hypertension, and coagulopathies, is even poorer than symptom recognition. This disparity between the high prevalence of traumatic injuries and the low degree of public knowledge is especially problematic, because early recognition of warning signals and proper immediate actions following injury can influence patient outcomes. When individuals are unaware of the signs of post-traumatic ICH or the importance of seeking prompt medical attention, opportunities for timely intervention may be missed, potentially leading to worse outcomes, including disability or death.

The treatment of TBI varies with injury severity, ranging from rest and pain medication for mild cases to oxygen support, blood pressure management, and perhaps surgery for moderate to severe injuries [3,13]. According to a systematic review, those who presented within 24 hours had a decreased prevalence of traumatic cerebral pathology; nevertheless, further study is needed to describe the effects of delayed presentation to the emergency department [14]. Few studies emphasized the importance of public awareness to recognize the early symptoms and hazards that might lead to appropriate medical care, considerably lowering the possibility of serious complications [13,15,16].

Given the high prevalence of RTAs in Saudi Arabia and the critical role of timely medical intervention in preventing mortality and long-term disability, it is important to understand the level of public awareness of post-traumatic ICH symptoms. Regional variations in socioeconomic status, access to healthcare education, and exposure to public health campaigns may result in different levels of awareness, which could impact pre-hospital decision-making and delay critical care. However, no similar studies have been conducted in other regions of Saudi Arabia. Therefore, this study aimed to assess public awareness and recognition of alarming symptoms of post-traumatic ICH in Taif, Saudi Arabia, including associated risk factors and pediatric-specific warning signs. The primary objective of this study was to assess the level of public awareness and recognition of alarming symptoms of post-traumatic ICH among adults in Taif, Saudi Arabia. The secondary objectives were to assess awareness of risk factors for post-traumatic ICH and compare awareness levels across sociodemographic characteristics, including age, sex, and education level; evaluate caregivers’ ability to recognize pediatric-specific warning signs of post-traumatic ICH; and describe participants’ care-seeking behavior following head trauma. It was hypothesized that the general population in Taif would demonstrate insufficient knowledge of the alarming symptoms of post-traumatic ICH, with significant variability based on demographic factors such as age, education level, and prior exposure to head trauma education.

## Materials and methods

Study design and sampling

This cross-sectional study was conducted in Taif, Saudi Arabia, between October 2025 and February 2026. Ethical approval was obtained from the Scientific Research Ethics Committee of Taif University (approval no. 47-041, dated 8 October 2025). A non-probability convenience sampling method was used to recruit the participants. Their inclusion and exclusion criteria are shown in Table 1. Recruitment was conducted primarily through WhatsApp community and interest groups representing diverse residential neighborhoods and demographic segments across Taif. Initial recipients were encouraged to further circulate the survey link within their own contacts (a snowball approach) to broaden reach beyond a single social network.

**Table 1:** Inclusion and exclusion criteria for participants

No.	Inclusion criteria	Exclusion criteria
1.	Adult aged ≥18 years (male and female)	Patients with severe illness
2.	Residing in Taif, Saudi Arabia	Have no internet connection
3.	Saudi and non-Saudi individuals	Health care workers

The sample size was calculated using the Raosoft sample size calculator (Raosoft Inc., Seattle, WA, USA) with a 5% margin of error, 95% confidence level, and 50% response distribution, yielding a minimum required sample of 385 participants. A total of 434 participants completed the survey and were included in the analysis. Written informed consent was obtained electronically at the beginning of the survey, with submission of the form serving as confirmation of voluntary participation. All questionnaire items were set as mandatory fields within Google Forms (Google LLC, Mountain View, CA, USA), so incomplete responses could not be submitted. To prevent duplicate entries, the survey was configured to permit only one submission per registered mobile phone number, restricting each participant to a single completed questionnaire.

Data collection

Data were collected using a previously published questionnaire that was obtained with permission via email from the original authors prior to data collection [15]. Since no previously validated Arabic-language questionnaire comprehensively covering the domains of interest was available, the questionnaire was designed based on an extensive review of the literature. The items on awareness are adapted from previous studies examining public knowledge of post-traumatic ICH symptoms, risk factors, and care-seeking behaviors [13,15,16]. The questionnaire was initially drafted in Arabic to ensure accessibility for the target population. To establish content validity, the preliminary version of the questionnaire was reviewed by a panel of three experts. Based on the experts' feedback, modifications were made to improve question clarity, remove ambiguous items, and ensure that all critical aspects of post-traumatic ICH awareness were adequately addressed. Following expert review, a pilot study was conducted to assess the questionnaire's clarity, comprehensibility, and practicality. The pilot sample consisted of 30 individuals recruited from the target population who were representatives of the public in Taif. Participants in the pilot were asked to complete the questionnaire and provide verbal feedback regarding any questions they found confusing, difficult to understand, or ambiguous. Based on the pilot participant feedback, minor modifications were made to the wording of several questions to enhance clarity. No major structural changes were required. Data from the pilot participants were not included in the final study sample.

Beyond internal consistency, content validity of the adapted questionnaire was established through the three-member expert panel review described above, in which items were rated for relevance, clarity, and coverage of the intended domains, supplemented by pilot participants’ feedback on face validity (clarity and comprehensibility of item wording). Internal consistency was subsequently assessed in the full study sample using Cronbach’s alpha via SPSS Statistics version 29 (IBM Corp., Armonk, NY, USA), and McDonald’s omega was calculated using the OMEGA macro for SPSS, both computed for the complete 16-item scale as a whole featured in the third section of the questionnaire. To evaluate each item’s contribution to overall scale reliability, item-deleted alpha and omega coefficients were also calculated for each item, reflecting the scale-level reliability that would result if that item were removed; these are diagnostic statistics rather than standalone reliability estimates for individual items, since reliability coefficients are properties of a scale rather than of a single item. Formal quantitative indices of content validity (e.g., a content validity index), corrected item-total correlations, and test-retest reliability were not calculated for the adapted instrument.

Moreover, to ensure transparency and reproducibility, the composite awareness score was calculated as the sum of 16 dichotomous items framed as (know, correct = 1) and (unknown, incorrect = 0), yielding a possible range of 0 to 16. Based on that, categorization of the score was done, and thresholds were defined as poor if the participants scored less than 50% of the questionnaire as correct and good awareness if they scored 50% or more as correct. This 50% threshold was adopted a priori as a conventional dichotomization point commonly used in awareness and knowledge survey research to classify composite scores into 'poor' versus 'good' categories in the absence of an externally validated clinical benchmark; it was pre-specified before data analysis rather than derived empirically from the study data. As this represents a relative rather than a clinically validated absolute standard, awareness scores categorized using this cut-off should be interpreted as indicative of comparative awareness levels rather than as a validated measure of adequate knowledge. A 50% cut-point is widely used in the knowledge, attitude, and practice (KAP) and health-awareness survey literature to distinguish 'poor' from 'adequate/good' performance on composite scores, typically in the absence of an externally validated clinical or psychometric threshold; the present study follows this established convention rather than proposing a novel cut-point. To ensure that this categorization did not obscure information, the continuous awareness score (mean ± SD) is reported alongside the categorical classification throughout the results section, and it is this continuous score, rather than the dichotomized variable, that was used for all sociodemographic and subgroup comparisons, providing a form of sensitivity check that does not depend on the choice of cut-point. A formal receiver operating characteristic (ROC)-based or distribution-based (e.g., latent class or median-split) derivation of the threshold, or a systematic comparison of results across multiple alternative cut-points, was not undertaken, because no external criterion measure of 'true' awareness was available against which to calibrate an optimal cut-point in this population

The questionnaire (consisting of five sections) was distributed through Google Forms on various social media platforms. The first section of the questionnaire was related to demographic data such as sex, age, family size, the number of children under 18, the number of elderly individuals over 60, marital status, education level, and the participants’ knowledge of how to interact with children, as well as the type of relationship they have with them. The second section of the questionnaire assessed participants’ awareness of potential risk factors associated with head trauma. These factors included hypertension, the use of blood-thinning medications, bleeding disorders, chronic hepatitis, and repeated minor head injuries. It also highlighted the importance of understanding the ‘lucid interval,' the period following the injury when symptoms might temporarily improve. The third section focused on the participants’ awareness of specific symptoms that require urgent medical attention, such as loss of consciousness or confusion shortly after the injury, persistent headache, decreased awareness or alertness (such as fatigue or difficulty waking up), loss of consciousness again, vision changes (e.g., double vision or pupil dilation), seizures, clear fluid draining from the nose or ears, slurred speech, weakness in the limbs or face, and balance issues. The fourth section evaluated the awareness and capability of participants, particularly mothers and caregivers, to recognize the concerning signs and symptoms following a head injury in children. It emphasized the importance of recognizing these symptoms to seek timely medical care. The concerning symptoms in children include changes in eating and feeding habits, persistent crying and irritability, difficulty concentrating, loss of interest in activities, changes in sleep patterns, loss of motor skills such as difficulty using the toilet or controlling bladder function, balance problems, dizziness, fatigue, and a decline in academic or cognitive performance. The final section specifically surveyed participants with a history of head trauma to assess how they responded after such injuries. They were asked about their previous experiences with head trauma (e.g., from falls or accidents) and were instructed to rate the severity of their injuries using a scale. Further questions determined whether the injury was accompanied by loss of consciousness, whether medical attention was sought, and whether the injury led to any symptoms mentioned earlier in the questionnaire. The complete English version of the questionnaire is provided in the appendices section (see Appendix A).

Data analysis

Data were analyzed using SPSS Statistics version 29. Descriptive statistics were presented as frequencies and percentages for categorical variables and as medians with interquartile ranges (IQR) for non-normally distributed continuous variables. Comparisons between participants who had experienced head trauma and those who had not were conducted using the chi-square test (χ²) or Fisher’s exact test, as appropriate, to assess associations between categorical variables. To compare awareness scores across different groups, both independent samples t-tests and one-way analysis of variance (ANOVA) were employed, depending on the number of groups being compared. Independent samples t-tests were used for comparisons between two groups, while ANOVA was applied when comparing more than two. Post hoc tests with appropriate corrections were conducted following ANOVA to identify specific group differences. The Shapiro-Wilk test and Levene's test for normality and homogeneity were used. No formal correction for multiple testing (e.g., Holm-Bonferroni or false discovery rate (FDR) adjustment) was applied; therefore, results should be interpreted cautiously due to the potential inflation of type I error. The composite awareness score was analyzed both as a continuous variable (mean ± SD) and as the pre-specified dichotomized (poor/adequate) category; continuous scores were used for all sociodemographic and subgroup comparisons (t-tests/ANOVA), while the dichotomized variable was used only for descriptive prevalence reporting so that categorization did not obscure the underlying distribution of awareness.

Moreover, multiple binary logistic regression was performed to identify demographic factors associated with awareness of post-traumatic ICH warning signs. For each one of the alarming signs examined, a separate logistic regression model was constructed with the dependent variable. The independent variables entered in each model were sex, age, and educational level. Sex was coded as a binary variable in every regression model, with female sex set as the reference category (0 = female, 1 = male); the reported odds ratios (OR) for sex therefore reflect the odds of a male participant correctly identifying a given symptom relative to a female participant. All predictors were entered simultaneously using the enter method. Model fit was assessed using the Hosmer-Lemeshow goodness-of-fit test, with a p-value greater than 0.05 indicating adequate fit. The overall significance of each model was evaluated using the Omnibus Tests of Model Coefficients, and the proportion of variance explained was estimated using Nagelkerke R². The results were presented as OR with 95% confidence intervals (CI) and corresponding p-values. A p-value of less than 0.05 was considered statistically significant.

As the sample size calculation (Raosoft) was designed to estimate the prevalence of awareness with adequate precision rather than to power the subgroup comparisons or logistic regression models, a post-hoc assessment of these analyses was performed. For the two-group comparisons (e.g., sex), the achieved sample of 434 participants provided approximately 80% power (two-tailed α = 0.05) to detect a minimum effect size of Cohen's d ≈ 0.27, corresponding to a small-to-medium effect; smaller true effects may therefore have gone undetected (type II error). For the logistic regression models, each of which included five parameters (sex, age, and three dummy-coded categories of educational level), the events-per-variable (EPV) ratio, calculated as the size of the smaller outcome category divided by the number of parameters, ranged from approximately 25 to 42 across the eight symptom-specific models, exceeding the conventionally recommended minimum of 10 EPV and supporting reasonably stable coefficient estimation despite the absence of an a priori power calculation for these models (Table 2).

**Table 2:** EP for the eight symptom-specific logistic regression models (total n = 434) *EPV=size of the smaller outcome category (unaware) ÷ number of parameters (k = 5: sex, age, and three dummy-coded educational-level categories); Conventionally recommended minimum EPV = 10 EPV: Events-per-variable

Alarming symptoms (logistic regression model)	Aware, n (%)	Unaware, n (%)	Parameters (k)	EPV *
Loss of consciousness or change in consciousness	305 (70.3)	129 (29.7)	5	25.8
Chronic headache	285 (65.7)	149 (34.3)	5	29.8
Decreased level of consciousness or alertness	260 (59.9)	174 (40.1)	5	34.8
Nausea or vomiting	285 (65.7)	149 (34.3)	5	29.8
Changes in vision (double vision/pupil dilation)	290 (66.8)	144 (33.2)	5	28.8
Convulsion	308 (71.0)	126 (29.0)	5	25.2
Clear fluid from nose or ears	225 (51.8)	209 (48.2)	5	41.8
Neurological signs (slurred speech, weakness, loss of balance)	280 (64.5)	154 (35.5)	5	30.8

## Results

Validation of the study questionnaire

The internal consistency of the 16-item questionnaire, treated as a single scale, was assessed using Cronbach’s alpha and McDonald’s omega. The overall Cronbach’s alpha and McDonald’s omega for the entire questionnaire were both 0.9, confirming excellent internal consistency and demonstrating that the questionnaire provides a robust and reliable measure of participants’ awareness of post-head injury symptoms. To examine each item’s contribution to this overall reliability, item-deleted alpha and omega coefficients were also calculated, indicating the scale-level reliability that would result if that item were removed (Table 2); these values ranged from 0.7 to 0.9 for alpha and from 0.7 to 0.9 for omega. Items related to chronic headache, convulsions, neurological symptoms, and vision changes showed the highest item-deleted reliability (α = 0.9, ω = 0.9), consistent with strong contributions to overall reliability, while items such as loss of consciousness or altered alertness, neurological signs, and vomiting/fatigue showed slightly lower item-deleted alpha values (α = 0.7) but maintained high item-deleted omega coefficients (ω = 0.9), indicating that removing any single item would not substantially improve overall reliability (Table 3).

**Table 3:** Item-deleted reliability analysis of the 16-item post-traumatic ICH awareness questionnaire, showing overall scale reliability (Cronbach’s alpha and McDonald’s omega) if each item were removed (total n = 434) Note: χ² = Pearson chi-square statistic (df = 1 for all comparisons); φ = phi coefficient (effect size for 2×2 tables; interpreted as negligible (<0.10), small (0.10-0.29), medium (0.30-0.49), or large (≥0.50). Test statistics were calculated from the reported group frequencies.

Question	Cronbach’s α if item deleted	McDonald’s ω if item deleted
Loss of consciousness or change in consciousness within minutes to hours after a head injury	0.7	0.9
Chronic headache	0.9	0.9
Decreased level of consciousness or alertness (e.g., feeling tired, difficulty waking up from sleep	0.8	0.9
Nausea or vomiting	0.9	0.7
Changes in vision such as double vision or dilation of one pupil	0.9	0.9
Convulsion	0.9	0.9
Clear fluid coming from the nose or ears	0.9	0.9
Neurological signs and symptoms include slurred speech, weakness in the arms, legs, or face, and loss of balance	0.7	0.9
Changes in eating and breastfeeding habits	0.9	0.9
Constant crying and distress	0.9	0.7
Changes in the ability to concentrate, and decreased interest in favorite games and activities	0.9	0.9
Change in sleep pattern	0.9	0.7
Loss of acquired primary skills, such as the ability to control urination and use the toilet	0.9	0.9
Inability to balance, unsteady gait	0.9	0.9
Vomiting and fatigue	0.7	0.9
Change or decline in academic performance	0.9	0.7

Baseline characteristics

A total of 434 participants from the Taif community were included in the study. Females comprised 68.2% (n = 296) of the sample, while males represented 31.8% (n = 138). Their median age was 29 years (IQR: 22-40). Most respondents (54.6%, n = 237) lived in families of five to eight members, and 72.4% (n = 314) reported having children under the age of 15, whereas 39.2% (n = 170) had elderly individuals above 60 years living in the same household. Regarding marital status, 52.5% (n = 228) were married, 43.5% (n = 189) were single, and 3.9% (n = 17) were divorced or widowed. The majority (69.8%, n = 303) held a higher education degree, and 80.6% (n = 350) reported frequently dealing with children (Table 4).

**Table 4:** Demographics characteristics of participants (total n = 434)

Parameters		n (%)
Family members	2-4	123 (28.3)
	5-8	237 (54.6)
	> 8	74 (17.1)
Presence of children <15 years	Yes	314 (72.4)
	No	120 (27.6)
Presence of elderly >60 years	Yes	170 (39.2)
	No	264 (60.8)
Marital status	Widowed	3 (0.7)
	Divorced	14 (3.2)
	Single	189 (43.5)
	Married	228 (52.5)
Educational level	Uneducated	3 (0.7)
	Educated (primary or intermediate)	19 (4.4)
	High school diploma	109 (25.1)
	High level of education	303 (69.8)
Frequency of dealing with children	I do not deal with children frequently	84 (19.4)
	In the workplace, for example: teaching, nursing, etc.	43 (9.9)
	A family member other than the mother	181 (41.7)
	Mother	126 (29.0)

Awareness of risk factors for post-traumatic ICH

A small proportion of participants (9.7%, n = 42) reported chronic diseases such as hypertension, hepatitis, or hemophilia as risk factors. Around 4.8% (n = 21) used anticoagulant medications. Only 21.4% (n = 93) recognized that comorbidities such as anticoagulant use, hemophilia, or hepatitis increase the likelihood of intracranial bleeding after head injury. About 46.5% (n = 202) showed awareness that repeated minor head trauma in the elderly could lead to post-traumatic ICH, and 42.4% (n = 184) knew that the post-seizure recovery period following a head injury requires hospital evaluation (Table 5).

**Table 5:** Awareness of risk factors for post-traumatic ICH (total n = 434) ICH: Intracranial hemorrhage

Parameter			n (%)
Presence of chronic diseases	Yes	Hypertension	36 (8.3)
		Hepatitis	4 (0.9)
		Hemophilia	2 (0.5)
	No		392 (90.3)
Use of anticoagulants	Yes		21 (4.8)
	No		413 (95.2)
Awareness that comorbidities (anticoagulants, hemophilia, hepatitis) increased risk	Yes		93 (21.4)
	No		341 (78.6)
Awareness that repeated minor head trauma in elderly may cause bleeding	Yes		202 (46.5)
	No		232 (53.5)
Awareness that post-seizure phase requires hospital evaluation	Yes		184 (42.4)
	No		250 (57.6)

Awareness of alarming symptoms of post-traumatic ICH that require hospital admission

Most participants knew several alarming symptoms of post-traumatic ICH. Specifically, 71.0% (n = 308) of the participants recognized that convulsion is an alarming sign, 70.3% (n = 305) knew that loss or change of consciousness indicates an alarming sign, and 66.8% (n = 290) recognized that changes in vision such as double vision or unequal pupil size are warning symptoms. Conversely, fewer participants recognized the less obvious signs (Table 6).

**Table 6:** Awareness of alarming symptoms of post-traumatic ICH that require hospital admission (total n = 434) ICH: Intracranial hemmarahge

Parameters		n (%)
Loss of consciousness or change in consciousness within minutes to hours after a head injury	Aware	305 (70.3)
	Unaware	129 (29.7)
Chronic headache	Aware	285 (65.7)
	Unaware	149 (34.3)
Decreased level of consciousness or alertness (e.g., feeling tired, difficulty waking up from sleep)	Aware	260 (59.9)
	Unaware	174 (40.1)
Nausea or vomiting	Aware	285 (65.7)
	Unaware	149 (34.3)
Changes in vision such as double vision or dilation of one pupil	Aware	290 (66.8)
	Unaware	144 (33.2)
Convulsion	Aware	308 (71.0)
	Unaware	126 (29.0)
Clear fluid coming from the nose or ears	Aware	225 (51.8)
	Unaware	209 (48.2)
Neurological signs and symptoms including slurred speech, weakness in the arms, legs, or face, and loss of balance	Aware	280 (64.5)
	Unaware	154 (35.5)

Awareness and ability to recognize alarming signs and symptoms of post-traumatic ICH in children

Regarding alarming signs in children, 68.4% (n = 297) of the participants identified inability to balance or unsteady gait, 68.2% (n = 296) recognized vomiting and fatigue, 47.7% (n = 207) were aware that changes in eating or breastfeeding habits may indicate brain injury, and 49.8% (n = 216) recognized that changes in sleep patterns can be an alarming symptom (Table 7).

**Table 7:** Awareness and ability to recognize alarming signs and symptoms in children (total n = 434)

Parameters		n (%)
Changes in eating and breastfeeding habits	Aware	207 (47.7)
	Unaware	227 (52.3)
Constant crying and distress	Aware	268 (61.8)
	Unaware	166 (38.2)
Changes in the ability to concentrate, and decreased interest in favorite games and activities	Aware	258 (59.4)
	Unaware	176 (40.6)
Change in sleep pattern	Aware	216 (49.8)
	Unaware	218 (50.2)
Loss of acquired primary skills, such as the ability to control urination and use the toilet	Aware	239 (55.1)
	Unaware	195 (44.9)
Inability to balance, unsteady gait	Aware	297 (68.4)
	Unaware	137 (31.6)
Vomiting and fatigue	Aware	296 (68.2)
	Unaware	138 (31.8)
Change or decline in academic performance	Aware	242 (55.8)
	Unaware	192 (44.2)

Total awareness scores and categorization of participants’ awareness levels

The total mean awareness score among participants was 9.8 ± 5.5, indicating a moderate level of overall awareness. When categorized, slightly more than half of the participants, 37.7% (n = 164), were classified as having poor awareness, while 62.2% (n = 270) demonstrated adequate awareness. These findings suggested that awareness within the study population is nearly evenly distributed, indicating the need for targeted interventions to improve knowledge and understanding among those with lower awareness levels (Table 8).

**Table 8:** Total awareness scores and categorization of participants’ awareness levels (total n = 434)

Variable	Value	
Total awareness score n (±SD)	9.8 ± 5.5	
Category of awareness n (%)	Poor awareness	164 (37.7)
	Adequate awareness	270 (62.2)

Participants’ reactions and experience with head Injury

Less than half (41.5%, n = 180) of the participants stated they would go to the hospital immediately, 38% (n = 165) would build their response on the severity and symptoms, and 19.4% (n = 84) would decide based solely on injury severity, regardless of symptoms. A total of 21.4% (n = 93) participants reported a previous head injury, with 48.4% (n = 45) rating the severity as mild (1-3/10), and 10.8% (n = 10) as severe (7-10/10). Among those with prior head injuries, 39.8% (n = 37) visited the hospital for evaluation. Approximately 41.9% (n = 39) reported that their injury was accompanied by one or more of the alarming symptoms (Table 9).

**Table 9:** Participants’ behavioral responses and experiences after head injury (total n = 434)

Questions/parameters	Response	n (%)
When you experience a head injury, what is your reaction?	Decide course of action depending on the severity of the injury, regardless of the symptoms	84 (19.4)
	Decide course of action depending on the severity and symptoms	165 (38)
	I will go to the hospital immediately	180 (41.5)
	Other	5 (1.2)
Do you have a history of head trauma, such as a fall, blow, or accident?	Yes	93 (21.4)
	No	341 (78.6)
Injury severity assessment	1-3	45 (48.4)
	4-7	38 (40.9)
	7-10	10 (10.8)
Visited hospital for evaluation after head injury	I suffered a head injury without losing consciousness	34 (36.6)
	No	22 (23.7)
	Yes	37 (39.8)
Trauma accompanied by any of the symptoms mentioned	Other symptoms	10 (10.8)
	No	44 (47.3)
	Yes	39 (41.9)

Comparison of awareness of alarming post-traumatic symptoms between participants with and without prior head trauma

Regarding awareness of alarming symptoms of ICH, no statistically significant differences were found between participants with and without a history of head trauma (p > 0.05 for all comparisons). Both groups showed similar levels of knowledge across all symptom categories, including loss of consciousness, headache, vomiting, neurological deficits, and fluid leakage (Table 10).

**Table 10:** Comparison of awareness of alarming post-traumatic symptoms of ICH between participants with and without prior head trauma (total n = 434) ICH: Intracranial hemorrhage

Parameters		History of head trauma, such as a fall, blow, or accident		χ² (df), effect size (φ), p-value
		Yes	No	
Loss of consciousness or change in consciousness within minutes to hours after a head injury	Aware	70 (75.3)	235 (68.9)	χ²(1) = 1.41, φ = 0.06; p = 0.252
	Unaware	23 (24.7)	106 (31.1)	
Chronic headache	Aware	69 (74.2)	216 (63.3)	χ²(1) = 3.82, φ = 0.09; p = 0.064
	Unaware	24 (25.8)	125 (36.7)	
Decreased level of consciousness or alertness (e.g., feeling tired, difficulty waking up from sleep)	Aware	63 (67.7)	197 (57.8)	χ²(1) = 3.02, φ = 0.08; p = 0.095
	Unaware	30 (32.3)	144 (42.2)	
Nausea or vomiting	Aware	65 (69.9)	220 (64.5)	χ²(1) = 0.94, φ = 0.05; p = 0.389
	Unaware	28 (30.1)	121 (35.5)	
Changes in vision such as double vision or dilation of one pupil	Aware	65 (69.9)	225 (66)	χ²(1) = 0.50, φ = 0.03; p = 0.535
	Unaware	28 (30.1)	116 (34)	
Convulsion	Aware	68 (73.1)	240 (70.4)	χ²(1) = 0.27, φ = 0.03; p = 0.699
	Unaware	25 (26.9)	101 (29.6)	
Clear fluid coming from the nose or ears	Aware	50 (53.8)	175 (51.3)	χ²(1) = 0.18, φ = 0.02; p = 0.726
	Unaware	43 (46.2)	166 (48.7)	
Neurological signs and symptoms include slurred speech, weakness in the arms, legs, or face, and loss of balance	Aware	61 (65.6)	219 (64.2)	χ²(1) = 0.06, φ = 0.01; p = 0.903
	Unaware	32 (34.4)	122 (35.8)	

Mean awareness scores across sociodemographic characteristics

The mean awareness scores varied across several sociodemographic and household characteristics. Males had a significantly lower mean awareness score (9.0 ± 6.2) compared to females (10.8 ± 5.5; p = 0.0063). Participants with children under 15 years also demonstrated higher awareness (10.7 ± 5.8) than those without (9.1 ± 5.7; p = 0.0056). Marital status was significantly associated with awareness, with widowed participants showing the lowest mean score (7.7 ± 8.6) and divorced participants who were the highest (13.2 ± 4.8; p = 0.0033). No significant differences were observed in awareness scores across family size (p = 0.58), presence of elderly members > 60 years (p = 0.11), or educational level (p = 0.99). Frequency of interaction with children was associated with awareness levels (p = 0.012), with participants who primarily cared for children as mothers showing the highest mean score (11.6 ± 5.4). These results indicated that sex, parental status, marital status, and direct interaction with children were significantly associated with awareness levels, while family size, presence of elderly members, and education level were not (Table 11). When a post-hoc Benjamini-Hochberg FDR correction was applied across these seven comparisons, the four significant associations survived correction (adjusted p-values: marital status = 0.015, presence of children <15 years = 0.015, sex = 0.015, and frequency of interaction with children = 0.021). All four also remained significant under the more conservative Holm-Bonferroni procedure, indicating that these sociodemographic associations with awareness are unlikely to reflect chance findings arising from the number of comparisons performed.

**Table 11:** The mean of awareness scores across sociodemographic characteristics (total n = 434)

Variable		Mean awareness score	p-value
Sex	Males	9.0 (6.2)	0.0063
	Females	10.8 (5.5)	
Family members	2-4	10.6 (5.8)	0.58
	5-8	10.0 (5.7)	
	> 8	10.4 (6.0)	
Presence of children <15 years	Yes	10.7 (5.8)	0.0056
	No	9.1 (5.7)	
Presence of elderly >60 years	Yes	10.8 (5.6)	0.11
	No	9.9 (5.9)	
Marital status	Widowed	7.7 (8.6)	0.0033
	Divorced	13.2 (4.8)	
	Single	9.2 (5.9)	
	Married	10.9 (5.6)	
Education level	Uneducated	10.0 (8.9)	0.99
	Educated (primary or intermediate)	9.9 (6.8)	
	High school diploma	10.3 (5.6)	
	High level of education	10.3 (5.8)	
Frequency of dealing with children	I do not deal with children frequently	9.1 (5.9)	0.012
	In the workplace, for example: teaching, nursing, etc.	9.9 (5.8)	
	A family member other than the mother	9.9 (5.9)	
	Mother	11.6 (5.4)	

The multiple binary logistic regression models showed that across all models, sex and age emerged as the most consistent predictors, while educational level was generally not associated with the awareness of alarming signs. Males demonstrated significantly higher awareness than females (i.e., OR greater than 1, with female sex as the reference category) for five symptoms including loss of consciousness (OR = 2.01, 95% CI (1.25-3.21), p = 0.004), nausea or vomiting (OR = 2.51, 95% CI (1.58-3.99), p < 0.001), vision changes (OR = 1.89, 95% CI (1.19-3.00), p = 0.007), convulsions (OR = 2.70, 95% CI (1.67-4.39), p < 0.001), and neurological signs (OR = 1.71, 95% CI (1.08-2.70), p = 0.022). When these five sex-predictor associations were evaluated as a family of eight symptom-specific comparisons, a post-hoc Benjamini-Hochberg FDR correction indicated that all five remained statistically significant (adjusted p-values ranging from <0.008 to 0.035). Under the more conservative Holm-Bonferroni procedure, four of the five associations remained significant (loss of consciousness, nausea/vomiting, vision changes, and convulsions). In contrast, the association with neurological signs (unadjusted p = 0.022) did not survive correction and should therefore be interpreted with additional caution. Age was a significant positive predictor in six models, with each one-year increase associated with approximately 2% to 3% higher odds of awareness. Educational level was significant only for convulsion awareness, where participants with a high school diploma had lower odds compared to those with higher education (OR = 0.36, 95% CI (0.13-0.96), p = 0.042). The models for chronic headaches, decreased consciousness, and clear fluid from nose or ears were not statistically significant, indicating that demographic factors did not predict awareness of these symptoms. The logistic regression models explained a relatively small proportion of the variance in outcomes, with R² values ranging from 4.7% to 9.2%, indicating limited explanatory power of the included variables. This suggests that additional factors beyond demographics contribute to public awareness of post-traumatic ICH alarming signs.

## Discussion

The current sample was predominantly young and relatively well-educated, which might be expected to have a good understanding of health issues and problems. This demographic distribution is essential because it suggests that most of the participants are from the active population that is likely to have a greater potential of facing head trauma incidents. However, there are still gaps in knowledge about risk factors and alarming symptoms that were identified, which aligns with international evidence that public awareness of TBI remains limited even in educated populations [13,17-19]. Furthermore, most participants recognized obvious red-flag symptoms such as convulsions and loss of consciousness, but awareness of more subtle or delayed manifestations (for example, visual changes or behavioral alterations) was lower. This pattern is consistent with broader TBI literature, where severe and visible neurological symptoms and signs are more likely to prompt concern, while internal or evolving symptoms are often underestimated by the public [20-22].

Moreover, only a minority of participants understood that comorbidities such as anticoagulant use, hemophilia, or liver disease increase the risk of post-traumatic bleeding, which was not supported by strong evidence that these factors are associated with higher rates of post-traumatic ICH and worse outcomes after head trauma [17-19]. Similar deficiencies in recognition of medical risk factors have been reported in studies examining public and patient knowledge in different regions of Saudi Arabia [15,16]. This suggests that there is a recurrent gap rather than a local phenomenon in Taif [16,21,23]. To add on, in view of the aging population and increasing use of anticoagulant medications worldwide, targeted education for patients and caregivers with these medications is important to increase their awareness in the setting of acute injuries [19,21].

The current participants’ awareness of alarming signs that require hospital admission was higher than that of a study conducted in Jeddah City (ranging from 71.0% to 51.8%). The study found that 49.6% of participants responded incorrectly to the alarming signs and symptoms (did not go to the hospital) because they were unaware [15]. This was also in agreement with previous research, which indicated that although post-traumatic ICH is usually related to head injuries, delayed interventions are rare. Furthermore, it is recommended that the severity of the findings on first brain imaging and serial clinical assessments should guide the requirement for follow-up imaging in the event of head trauma [24].

A key novel contribution of this study is the explicit comparative analysis of post-traumatic ICH awareness levels between our Taif sample and other major regions of Saudi Arabia, including Riyadh and Jeddah. While previous research has largely examined awareness in isolated geographic or clinical samples, our study directly contextualizes Taif-specific findings against regional benchmarks derived from recent cross-sectional studies. For instance, we observed that although overall awareness in our sample was moderate, certain knowledge gaps were more pronounced in our sample compared to Riyadh, whereas awareness of early warning symptoms was relatively higher in our sample than in Jeddah. This regional comparison not only highlights heterogeneous patterns of post-traumatic ICH awareness within the Saudi kingdom but also underscores the importance of tailoring public health interventions to local sociodemographic and cultural contexts. To our knowledge, this is among the first studies to systematically benchmark awareness in Taif against other Saudi populations, thereby enhancing the geographic novelty and practical relevance of the findings. However, because all three studies relied on non-probability convenience samples recruited through different methods and time periods, these cross-city comparisons are descriptive rather than based on a formal statistical test of population differences, and the awareness levels reported for our sample should not be extrapolated as representative of the general population of Taif.

Nonetheless, pediatric presentations of post-traumatic ICH represent another critical area that is mostly not seen, especially by the public. In the current study, results have shown that caregivers commonly recognized obvious symptoms such as vomiting (68.2%) or unsteady gait (68.4%), and fewer identified changes in feeding (47.7%) or sleep (49.8%) as potential alarming signs of post-traumatic ICH in children. These results were not related to the current local settings, as pediatric head injury research highlights that subtle behavioral and sleep disturbances may be the earliest manifestations of intracranial pathology in young children and are often missed by caregivers [25,26]. In the same context, a study conducted in Western Saudi Arabia revealed a deficiency in understanding of the management of head trauma and avoiding home injuries in children among mothers [27]. So, early recognition of these signs is essential because delayed diagnosis in pediatric TBI and post-traumatic ICH has been associated with worse neurodevelopmental and functional outcomes [25,28].

The current results showed that 41.5% of the participants should go to the hospital immediately as a necessary action when experiencing a child’s head injury, which was higher than some studies’ values that were conducted in the Western Region of Saudi Arabia. A study conducted in Madinah City, Saudi Arabia, reported that almost one-third of the parents had proper knowledge, while most parents had improper knowledge regarding first-aid management of epistaxis due to trauma [29]. However, in Riyadh, around 16.5% of parents had a high level of awareness of the necessary action following head trauma by keeping the child in a sitting position with the head slightly backward due to epistaxis [30]. Another study was undertaken in Makkah, Saudi Arabia, to measure mothers' KAPs on child-related house accidents. The results revealed that around 36% of mothers had poor knowledge, 38% had a positive attitude, and one-third recorded appropriate practice regarding child home accidents [31].

Only 41.5% of the current participants showed that they would go to the hospital immediately after a head injury. However, the care-seeking behavior observed in the present study, where most of the participants would wait for symptom progression or rely on self-judgment and approximation of injury severity, agrees with the international and local reports that many individuals with mild head injuries do not seek immediate medical evaluation [20,21,32]. Such delays, even in mild cases, may be concerning, given growing evidence that even mild post-traumatic ICH can potentially lead to significant cognitive, emotional, and quality-of-life consequences in some patients [28,32,33]. Nonetheless, interventions that improve knowledge about when to seek emergency assessment after head trauma have been shown to increase the likelihood of appropriate care-seeking behavior and may be highly relevant in those types of injuries [32].

Moreover, the absence of significant differences in awareness between participants with and without prior head trauma suggests that personal experience alone does not guarantee improved knowledge about TBI. This finding could also be explained by the possibility that most prior injuries in this sample were mild, and individuals with more severe post-traumatic ICH, who might have different awareness levels, were less likely to participate due to mortality or disability. Similar findings have been noted in multiple different studies where previous TBI injury did not consistently translate into better understanding of TBI or adherence to follow-up or recommendations, thus suggesting the importance of structured education at the point of care before discharging the patient from hospital care [13,23,28,33]. However, Alqarni et al. [13] reported that the people living in Jeddah city with a history of previous head trauma showed slightly higher awareness of decreased consciousness, which may reflect their personal experiences. This disparity reflects a gap in knowledge in different regions of Saudi Arabia. So, standardizing counseling about risk factors, red-flag symptoms, and indications for urgent reassessment in emergency department and primary care encounters could help transform individual injury episodes into opportunities for community-level education [16,23].

Furthermore, the present study demonstrated that awareness levels varied across several sociodemographic characteristics. Female participants exhibited significantly higher awareness scores compared to males, which may be partially explained by the higher proportion of females in the sample and their potentially greater involvement in childcare-related activities. Similarly, participants with children under 15 years and those who reported frequent interaction with children, particularly mothers, showed higher awareness levels, which suggests that direct caregiving responsibilities may enhance recognition of symptoms. Marital status was also associated with awareness, although the variability observed between categories should be interpreted cautiously given potential sample size imbalances. In contrast, no significant differences were identified across educational levels, family size, or the presence of elderly household members, which may indicate that awareness is influenced more by practical exposure and caregiving roles rather than formal education alone. However, these findings should be interpreted with caution due to the study’s sampling approach and potential residual confounding. The predominance of female participants and the use of convenience sampling may have influenced the observed associations, limiting their generalizability. Additionally, given the relatively modest differences in mean scores and the cross-sectional design, causal inferences cannot be established. Further studies using more representative samples are needed to better understand the determinants of awareness.

However, the present findings showed that male sex and older age were associated with higher odds of awareness of several individual post-traumatic ICH alarming signs in the symptom-specific regression models. Notably, this finding appears to contrast with the composite awareness scores, where female participants scored significantly higher overall; this apparent inconsistency likely reflects the difference between an aggregate composite measure and covariate-adjusted, symptom-specific associations and should not be interpreted as contradictory. A plausible explanation is that males in this sample were more likely to correctly identify certain individual and often more overt, alarming symptoms (e.g., convulsions, loss of consciousness, vision changes), which drove the higher odds observed for males in the symptom-specific regression models, while at the same time recognizing fewer of the full set of the 16 items overall, which lowered their mean composite awareness score relative to females. Because the composite score aggregates correct responses across all items irrespective of which specific symptoms are known, a group can show higher odds of recognizing several individual symptoms while still obtaining a lower total score if it recognizes fewer symptoms overall; these two summary measures are therefore not directly comparable and can legitimately point in different directions within the same sample.

Given the use of convenience sampling and the absence of correction for multiple comparisons across the individual symptom models, these sex- and age-related associations should be interpreted with caution rather than as robust or generalizable predictors. A post-hoc Benjamini-Hochberg FDR correction applied across the eight symptom-specific models indicated that four of the five significant sex-predictor associations also survived the more conservative Holm-Bonferroni correction, suggesting that these particular findings are unlikely to be solely an artifact of multiple testing. This aligns with broader research on health knowledge, where males often show higher awareness of certain symptoms, and older age correlates with increased recognition of warning signs. For example, studies on stroke awareness have found that higher education and prior experience with the condition improve knowledge, but sex and age remain important factors influencing awareness levels [34,35]. Moreover, the limited role of educational level in most models except convulsion awareness is consistent with research showing that education’s impact can vary depending on specific symptom or condition [36]. The lack of demographic prediction for some symptoms suggests that other factors such as personal experience, cultural influences, or targeted health education may play a significant role in public knowledge [37]. The relatively low variance explained by demographic factors indicates the need to explore additional determinants like health literacy, access to information, and community outreach to better understand and improve awareness of post-traumatic ICH warning signs. Overall, these results highlight the importance of tailoring public health interventions by considering demographic differences while addressing broader educational gaps.

Based on the specific knowledge gaps identified in this sample, the following targeted public health strategies may be considered as preliminary, hypothesis-generating suggestions rather than validated policy directives. First, patients prescribed anticoagulants should receive counselling at the time of dispensing, emphasizing the need for immediate evaluation following any head trauma, regardless of severity. Second, to address low awareness of mild pediatric symptoms, illustrated alarming signs could be distributed to all new parents. Public service announcements through social media platforms in Saudi Arabia should demonstrate clear 'what to do' scenarios after a head injury. Third, emergency departments could provide brief standardized discharge brochures covering risk factors, alarming signs, and indications for return. These suggestions respond to the specific deficiencies identified in our convenience sample and are intended as a starting point for local piloting rather than as generalizable, ready-to-implement public health policy. Because participants were recruited through non-probability convenience sampling and are not representative of the general population of Taif, we deliberately avoid framing these as population-level policy recommendations; before any broader implementation or policy adoption, their feasibility and effectiveness should be evaluated in future studies employing probability-based or multi-modal recruitment strategies that better represent the demographic groups underrepresented here, such as older adults and individuals with lower digital literacy or educational attainment.

Although this study could be the first on the topic in Taif City, it still has several limitations. A cross-sectional survey from a single city limits generalizability to other cities in the same region (Western region). Also, self-reported awareness and behavior are subject to recall and social desirability bias, and the use of a questionnaire cannot verify whether participants would act as reported in real head injury situations or not. Although the questionnaire underwent expert panel review, pilot testing, and internal consistency assessment, formal quantitative indices of content validity (e.g., a content validity index) and test-retest reliability were not calculated, so its psychometric validation remains incomplete beyond reliability assessment alone. In addition, corrected item-total correlations and a formal exploratory or confirmatory factor analysis were not performed; the 16 items were instead treated as a single unidimensional awareness checklist by design, with each item contributing one point to a summed composite score based on the content validity established through expert panel review, rather than on statistically derived dimensionality. Given the high overall and item-deleted reliability coefficients, a single underlying dimension is plausible, but this was not formally tested, and future validation work should incorporate item-total correlations and factor-analytic confirmation of the scale’s structure. Similarly, the 50% threshold used to dichotomize composite awareness scores, although pre-specified a priori and consistent with conventions used in comparable survey research, was not derived from an externally validated clinical benchmark; this arbitrary cut-off should therefore be interpreted with caution when comparing awareness prevalence across studies. To limit the potential loss of information inherent to dichotomization, the continuous awareness score (mean ± SD) was reported alongside the categorical classification (Table 8) and was the outcome used for all sociodemographic comparisons (Table 11), rather than the dichotomized variable, which was retained only for descriptive prevalence reporting.

A formal sensitivity analysis re-deriving the threshold via a receiver operating characteristic (ROC)-based or distribution-based approach, or systematically comparing results across a range of alternative cut-points, was not performed, as no externally validated clinical or psychometric benchmark for post-traumatic ICH awareness exists against which such a threshold could be calibrated in this population; future studies using criterion-validated instruments could apply ROC or latent-class methods to derive an empirically justified cut-point. In addition, selection bias may exist if individuals with higher health interests or education were more likely to participate, potentially overestimating true community awareness. Finally, the study did not correlate awareness with actual clinical outcomes or healthcare utilization. So, the direct impact of these knowledge gaps on morbidity and mortality from post-traumatic ICH cannot be determined. To add, the separate logistic regression models were used to allow for symptom-specific analysis, consistent with previous studies examining predictors of individual symptoms.

However, given the use of multiple models, the results should be interpreted cautiously due to the potential inflation of type I error; a post-hoc multiplicity-correction sensitivity analysis addressing this concern is reported below. It is also important to note that the sample size calculation was based on a 50% prevalence assumption and was powered only to estimate prevalence with adequate precision; it was not calculated to power the multiple logistic regression models, subgroup comparisons, or ANOVA-based hypothesis tests reported elsewhere in this manuscript. These analyses should therefore be considered exploratory rather than confirmatory, and the possibility of type II error (failure to detect true associations) cannot be excluded. Future studies should apply an appropriate correction for multiple comparisons, such as the Benjamini-Hochberg FDR procedure, to strengthen the statistical validity of the reported associations. The relatively low R² values (4.7-9.2%) further suggest that other unmeasured factors may contribute to these outcomes, highlighting the need for further research to identify additional predictors. Regarding statistical power, a post-hoc calculation indicated that the achieved sample provided approximately 80% power to detect small-to-medium effects (Cohen's d ≈ 0.27, two-tailed α = 0.05) in the main two-group comparisons, while the events-per-variable ratio for the logistic regression models (five parameters per model: sex, age, and three educational-level dummy categories) ranged from approximately 25 to 42, exceeding the conventional minimum of 10 EPV recommended for stable estimation. Although these figures suggest that the sample was reasonably adequate for the analyses performed, the possibility of undetected smaller effects, particularly for the subgroup and ANOVA-based comparisons with unequal or smaller group sizes (e.g., marital status categories), cannot be excluded.

Furthermore, the use of convenience sampling through online platforms represents a significant methodological limitation. This recruitment strategy inherently introduces selection (volunteer) bias, as it systematically excludes individuals with limited internet access, lower digital literacy, or those who do not use social media platforms. Given that the study population was predominantly young and well-educated, the present sample cannot be considered representative of the broader population of Taif, particularly older adults, individuals with lower educational attainment, and those from underserved communities. This is especially relevant because older adults are at higher risk for post-traumatic ICH and its complications, particularly those on anticoagulant therapy, yet they may be precisely the population underrepresented in our sample. Consequently, the current findings may overestimate the true level of public awareness in the general population, as individuals with higher education and health literacy were more likely to participate. Future studies should employ probability sampling methods and multi-modal recruitment strategies (including telephone surveys, community center outreach, and healthcare facility-based recruitment) to obtain a more representative sample and more accurately characterize awareness across all demographic groups in Taif. Overall, these findings support that public awareness regarding risk factors, signs and symptoms, and health-seeking behavior for post-traumatic ICH remains insufficient despite the growing global and regional burden of TBI [16,17,20,21]. These findings suggest that community-focused educational campaigns, school- and parent-targeted programs on pediatric head injury, and systematic patient counseling in clinical settings could potentially improve early recognition. However, interventional studies are needed to confirm whether such strategies would effectively reduce the preventable morbidity associated with delayed post-traumatic ICH diagnosis in this population [21,23,25,26,28].

## Conclusions

This study identified a moderate level of recognition for selected symptoms, particularly altered levels of consciousness and convulsions, while awareness of several risk factors and child-specific symptoms remained limited, including changes in feeding habits, breastfeeding patterns, and sleep routines. Although male sex and older age showed significant associations with awareness of certain individual post-traumatic ICH warning signs, these findings warrant cautious interpretation because of the study’s convenience sampling, cross-sectional design, and lack of adjustment for multiple comparisons. As the study relied on a non-probability convenience sample recruited primarily through social media, these estimates cannot be extrapolated to the general population of Taif and should instead be regarded as indicative of the awareness levels of this specific, largely young, well-educated, and predominantly female sample of social media users. These findings highlight the need for targeted public education initiatives, such as awareness programs in schools, universities, and community settings, as well as the dissemination of educational materials in healthcare facilities. While improving public recognition of red-flag symptoms may be beneficial, this study did not assess clinical outcomes; therefore, any potential impact on morbidity or mortality remains speculative and warrants further investigation in future research.

## Appendices

Appendix A

The complete English version of the questionnaire distributed to participants of this study is presented below.

Section One: Participant Demographic and Basic Information

1. Gender: ☐ Male ☐ Female

2. Age: ………

3. Number of Family Members:

☐ 2-4

☐ 5-8

☐ More than 8

4. Are there any children under the age of 15 in your household?

☐ Yes

☐ No

5. Are there any older adults (aged 60 years or above) in your household?

☐ Yes

☐ No

6. Marital Status:

☐ Single

☐ Married

☐ Divorced

☐ Widowed

7. Educational Level:

☐ Uneducated

☐ Educated (primary or intermediate)

☐ High school diploma

☐ High level of education

8. Frequency of Contact with Children:

☐ I do not deal with children frequently

☐ In the workplace, for example: teaching, nursing, etc

☐ As a family member (other than the mother).

☐ Mother

Section Two

The following questions aim to assess your awareness of the risk factors that cause brain hemorrhage following head injuries.

1.     Do you have any chronic disease?

☐ Yes    ☐ No

If yes, please specify:

Hypertension

Hepatitis

Hemophilia

2.     Do you use anticoagulant medications?

☐ Yes  ☐ No

3.     Do you think that comorbidities such as anticoagulant use, hemophilia, or hepatitis increase the risk of intracranial bleeding after head injury?

☐ Yes    ☐ No

4.     Do you think that repeated minor head trauma in the elderly may cause intracranial bleeding?

☐ Yes    ☐ No

5.     Do you think that the post-seizure phase after head injury requires hospital evaluation?

☐ Yes    ☐ No

Section Three: Awareness of Alarming Symptoms of Post-Traumatic Intracranial Hemorrhage (ICH) Requiring Hospital Admission

Do you know that loss of consciousness or a change in consciousness within minutes to hours after a head injury is an alarming symptom? ☐ Know ☐ Don’t Know

Do you know that chronic headache is an alarming symptom after a head injury? ☐ Know ☐ Don’t Know

Do you know that a decreased level of consciousness or alertness (e.g., feeling tired or difficulty waking up from sleep) is an alarming symptom after a head injury? ☐ Know ☐ Don’t Know

Do you know that nausea or vomiting is an alarming symptom after a head injury? ☐ Know ☐ Don’t Know

Do you know that vision changes, such as double vision or dilation of one pupil, are alarming symptoms after a head injury? ☐ Know ☐ Don’t Know

Do you know that convulsions are an alarming symptom after a head injury? ☐ Know ☐ Don’t Know

Do you know that clear fluid coming from the nose or ears is an alarming symptom after a head injury? ☐ Know ☐ Don’t Know

Do you know that neurological signs and symptoms such as slurred speech, weakness in the arms, legs, or face, and loss of balance are alarming symptoms after a head injury? ☐ Know ☐ Don’t Know

Section Four: Awareness and Ability to Recognize Alarming Signs and Symptoms in Children

Do you know that changes in eating and breastfeeding habits may be a warning sign after a head injury? ☐ Know ☐ Don’t Know Do you know that constant crying and distress may be a warning sign after a head injury? ☐ Know ☐ Don’t Know Do you know that changes in the ability to concentrate or decreased interest in favorite games and activities may be a warning sign after a head injury? ☐ Know ☐ Don’t Know Do you know that changes in sleep patterns may be a warning sign after a head injury? ☐ Know ☐ Don’t Know Do you know that loss of previously acquired skills, such as the ability to control urination or use the toilet, may be a warning sign after a head injury? ☐ Know ☐ Don’t Know Do you know that inability to balance or an unsteady gait may be a warning sign after a head injury? ☐ Know ☐ Don’t Know Do you know that vomiting and fatigue may be warning signs after a head injury? ☐ Know ☐ Don’t Know Do you know that a change or decline in academic performance may be a warning sign after a head injury? ☐ Know ☐ Don’t Know

Section Five: Participants’ Behavioral Responses and Experiences After Head Injury

1.  If you experience a head injury, what would be your reaction? ☐ Depending on the severity of the injury, regardless of the symptoms ☐ Depending on the severity of the injury and the symptoms ☐ I will go to the hospital immediately ☐ Other 2. Have you ever had a head injury, such as a fall, blow, or accident? ☐ Yes ☐ No 3. If you have experienced a head injury, how would you rate its severity? ☐  (1-3) ☐  (4-7) ☐  (8-10) 4. Did you go to the hospital after the head injury? ☐ Yes ☐ No ☐ I suffered a head injury without losing consciousness 5. Was the head injury accompanied by any of the symptoms mentioned previously? ☐ Yes ☐ No ☐ Other symptoms
